# Association of triglyceride-glucose index with vascular risk factors and clinical outcomes among COVID-19 patients: a retrospective cross-sectional study in Mengo Hospital, Kampala, Uganda

**DOI:** 10.11604/pamj.2023.46.113.41795

**Published:** 2023-12-21

**Authors:** William Lumu, Ronald Kasoma Mutebi, Susan Nakireka, David Muyanja, Simon Eleku, Denis Kaddu, Ezra Nunda, Deus Kabugo, Henry Kinene, Simon Nambago, Caroline Ninsiima, Isa Kifuba, Deogratious Edemaga, Edgar Atwiine, Brian Mutebi, Majorine Nandawula, Noeline Nakigudde, Zubeda Kataike, Jackline Nakachwa, Catherine Nakaayi, Paul Lukyamuzi, Robinson Ssebuufu, Rose Mutumba

**Affiliations:** 1 Department of Internal Medicine, Mengo Hospital, Kampala, Uganda

**Keywords:** Cardiovascular, risk, factors, clinical, outcomes

## Abstract

**Introduction:**

triglyceride-glucose (TyG) index is a reliable surrogate marker of insulin resistance. We assessed the association between triglyceride-glucose (TyG) index and vascular risk factors and clinical outcomes of critically ill adult COVID-19 patients.

**Methods:**

data from the charts of all patients with a confirmed diagnosis of COVID-19 who were hospitalized at Mengo Hospital Uganda from December 2020 to August 2021 was used for this study. Data on demographics, past medical history, clinical presentation, laboratory findings and clinical outcomes within the first 10 days of admission was extracted. TyG index was calculated as Inverse (triglyceride (mg/dl) x fasting glucose level (mg/dl)/2 and defined vascular risk factors using standard methods. Bivariate and multivariate logistic regression was conducted to establish a significant association. Statistical significance was set at p< 0.05.

**Results:**

out of 314 patients, 176 (56%) were females. The mean age ± SD was 58.2 years ± 16.82. The median TyG index was 9.76 (9.29-10.33). A high TyG index was found among 85.4% (n= 268, 95% CI: 0.809-0.889) of patients. Elevated total cholesterol was in 55.4% (n=174), triglycerides 70.7% (n=222), LDL 64.7% (n=203), blood glucose 80.6% (n=253), systolic blood pressure 43% (n=135) and 24.8% (n=78) diastolic blood pressure. The majority 49.7% ( n=156) were discharged, 22.0% (n=69) needed admission to the intensive care unit (ICU), 15.3% (n=48) died in the unit and 13.0% (n=41) had a composite outcome. The TyG index was significantly associated with glycated hemoglobin (AOR=1.029, 95%CI 0.561-1.496, p<0.001), low-density lipoprotein cholesterol (AOR=0.121,95%CI 0.023-0.219, p=0.016), high-density cholesterol (AOR=1.956, 95%CI 1.299-2.945, p=0.001), total cholesterol (AOR=2.177, 95%CI 1.5222-3.144, p<0.001, hospital death (AOR=0.778, 95%CI 0.623-0.972, p=0.028) and composite outcome (AOR=1.823, 95% CI 1.221-2.559, p=0.023). There was no association between hypertension and TyG index.

**Conclusion:**

a high TyG index was associated with vascular risk factors and clinical outcomes.

## Introduction

In the winter of 2019, the first human infection of Severe Acute Respiratory Syndrome Coronavirus 2 (SARS-CoV-2) was documented in Wuhan, China. One of its varied presentations is aggressive pneumonia which is associated with profound morbidity and mortality. SARS-CoV-2 the causal agent of Corona Virus Disease of 2019 (COVID-19) spread globally at a terrific speed and in pandemic proportions stretching medical care systems worldwide [1]. Earlier epidemiological studies showed a high propensity of COVID-19-related complications among individuals with pre-existing comorbidities [2]. Individuals with hypertension, diabetes, cardiovascular disease and obesity tend to suffer from severe pneumonia with poor clinical outcomes compared to those without [3]. Among these comorbidities, type 2 diabetes has been frequently shown to be a major predictor of poor clinical outcomes among COVID-19 patients [4]. Type 2 diabetes is often associated with insulin resistance [5].

In addition to type 2 diabetes, insulin resistance is associated with hypertension, dyslipidemia, cardiovascular disease, malignancy, obesity, neurodegenerative, inflammatory and infectious diseases [2]. All the disease conditions linked with insulin resistance have been associated with severity and poor clinical outcomes in critically ill patients including COVID-19. One of the resultant effects of insulin resistance is hyperinsulinemia [6]. When macrophages are chronically exposed to excessive levels of insulin, there is ensuing production of pro-inflammatory cytokines and ill-response to the pathogens [2]. Therefore, insulin resistance is associated with a hyper-activated inflammatory response that results in a cytokine storm which is a pathogenic orchestrator of critical illness and poor clinical outcomes in critical illnesses such as COVID-19 [7].

The gold standard for the assessment of insulin resistance is the hyperinsulinaemic-euglyceamic clamp (HIEC) but this is not usually feasible in real practice because of cost and its complexity [8]. However, the Homeostasis Model Assessment of Insulin Resistance (HOMA-IR) is the most often used method. HOMA-IR is of limited utility in patients receiving insulin treatment, those without functioning β-cells and its requirement of circulating insulin which is not routinely measured [8,9]. Thus, insulin resistance can be reliably and accurately measured by the triglyceride-glucose (TyG) index [8].

A high TyG index has been associated with diabetes, metabolic syndrome, hypertension, cardiovascular disease and severe COVID-19 infections and or mortality [8,10]. The relationship of TyG index as a surrogate marker of insulin resistance between cardiovascular risk factors and clinical outcomes among critically ill patients such as COVID-19 patients have been investigated in Asia and other regions of the world [2,11,12]. This important relationship is not known in our setting. Therefore, the current study was conducted to evaluate the association between the triglyceride-glucose index and vascular risk factors and clinical outcomes among adult COVID-19 patients admitted to Mengo Hospital.

## Methods

Study design and setting: this retrospective cross-sectional study was conducted at Mengo Hospital, Kampala, Uganda and involved review of medical charts of COVID-19 patients admitted with moderate to critical disease [13] to the hospital COVID-19 Treatment Unit (CTU) from December 2020 to August 2021. The study involved extracting data from the charts of COVID-19 patients who were admitted to Mengo Hospital CTU. The facility had 48 beds and was accredited by the Ministry of Health to provide treatment for moderate to severe cases of COVID-19. In this unit, national guidelines were followed to diagnose COVID-19 and profile patients for admission.

Study population: we obtained data from charts of patients hospitalized with a confirmed diagnosis of COVID-19 with moderate to critical disease [13] from December 2020 to August 2021. In the Mengo Hospital CTU, COVID-19 was diagnosed using polymerase chain reaction (PCR) and SARS-CoV-2 rapid antigen test with or without compatible chest imaging.

The formula for the cross-sectional survey was used to estimate the number of charts required [14]:


$$Sample\;size=\frac{Z^{2}P\left(1-P\right)}{d^{2}}$$

where Z=1.96 at α = 0.05 level of significance, p is the proportion of elevated TyG index in COVID-19 patients in a previous study and d = 0.05 the absolute error or precision. Basing on the study done by Ren H *et al*. 22% of COVID-19 patients who died had elevated TyG index [12]. Therefore, the sample size was:


$$Sample\;size=\frac{Z^{2}P\left(1-P\right)}{d^{2}}$$

The number was increased by 20% to cater for the dropping of files with more than 75% of missing data. We thus intended to obtain data from 317 charts. However, out of the 500 available charts, we were only able to select 314 charts that met our inclusion criteria. The charts were consecutively selected.

**Inclusion criteria:** all patients´ data hospitalized with a confirmed diagnosis of COVID-19 in the study period were included.

**Exclusion criteria:** we excluded patients´ charts that lacked data on fasting blood glucose (FBG), lipid profile and glycated hemoglobin (HbA1c) and those who were referred to other facilities, as we could not ascertain their clinical outcomes.

The dependent variable (primary outcome) was the triglyceride-glucose index (TyG) index which was derived from the inverse (fasting triglycerides (mg/dl) x fasting glucose (mg/dl)/2) and this was categorized into low and high cardiovascular risk [15]. The independent variables included age, sex, medical condition history, medicine use history, oxygen saturation, temperature, pulse, blood pressure, glycated heamoglobin, total cholesterol, low-density cholesterol, non-high-density cholesterol D-dimers, C-reactive protein, urea, creatinine, electrolytes, transaminases, total white cell count, neutrophil count, lymphocyte count, eosinophil count, platelet count, chest computed tomography (CT) scan findings and clinical outcomes.

**Laboratory analysis:** the Mengo Hospital CTU protocol for sample collection was followed to obtain samples. Within the unit, blood samples were collected in the morning 8 hours after the last oral intake. The samples were aseptically collected by venipuncture of the brachial vein in a 5 ml ethylenediaminetetraacetic acid (EDTA) tube and a 5 ml plain tube and these were immediately transported to the Mengo Hospital main laboratory for analysis. Plasma and serum specimens were separated by centrifugation at 3000 r/min for immediate analyses. Fasting blood glucose, glycated hemoglobin, total cholesterol (TC), high-density cholesterol (HDL), triglycerides (TG), low-density cholesterol, C-reactive protein, electrolytes, creatinine, urea, liver enzymes, d-dimers, complete blood count were measured by photometric assays and ion selective electrode measurement using the Roche Hitachi Cobas C311 chemistry analyzer as described elsewhere [16]. The erythrocyte sedimentation rate (ESR) was determined by Westergreen method [17].

**Data collection:** trained research assistants extracted retrospective data from patients´ charts admitted to the Mengo Hospital CTU from December 2020 to August 2021 and recorded it in the data extraction tool. Data on demographics (age, sex), medical conditions (diabetes, hypertension, stroke, chronic liver disease and Human Immune-deficiency Virus (HIV), medication history, clinical symptoms, and signs). Data on temperature, pulse, oxygen saturation, and blood pressure on admission were extracted. The EDAN vital signs monitor machine model M3 manufactured by EDAN Instruments Inc. China was used in the COVID-19 Treatment Unit to measure temperature, pulse, oxygen saturation, and blood pressure. We recorded laboratory findings on fasting blood glucose, glycated hemoglobin, total cholesterol (TC), high-density cholesterol (HDL), triglycerides (TG), low-density cholesterol (LDL), non-high-density cholesterol (non-HDL) C-reactive protein (C-RP), erythrocyte sedimentation rate (ESR) electrolytes, creatinine, urea, liver enzymes, D-dimers, complete blood count and chest computerized scan findings. We extracted data on clinical outcomes up to 10 days of admission.

### Definitions

**Triglyceride-Glucose Index (TyG):** was derived using a recently validated formula: inverse [fasting triglycerides (mg/dl) x fasting glucose (mg/dl)/2] [18]. We took cut off values of less than 9.04 and higher than 9.04 to denote low TyG and high TyG indices respectively [15]. In a study by Araújo SP *et al*. 2022, TyG values ≥9.04 were associated with cardio-metabolic factors such as total cholesterol (TC) low-density lipoprotein cholesterol (LDL), very low-density lipoprotein cholesterol (VLDL), uric acid, alananie aminotransferase (ALT), aspartate aminotransferase (AST), waist-hip ratio, systolic blood pressure (SBP), HOMAIR, smoking, metabolic syndrome, diabetes, and hepatic steatosis [15].

Vascular risk factors were defined as factors that traditionally cause vascular disease through endothelial dysfunction [19,20]. The vascular risk factors were hypertension defined as a systolic blood pressure ≥140mmHg or diastolic blood pressure ≥90mmHg [21] or history of hypertension on ant hypertensive medications as recorded in the patients' chart, dyslipidemia defined as a total cholesterol of ≥5.13mmol/l (≥200mg/dl) or LDL ≥2.82mmol/l (≥110mg/dl) or high density lipoprotein cholesterol (HDL) <1.02 (<40mg/l), non-HDL ≥3.33mmol/l (≥l30mg/dl), or triglyceride (TG) ≥1.68mmol/l (≥150mg/dl) or being on lipid-lowering medications [22]. Hyperglycemia was categorized as; diabetic patients who were either individuals with a prior history of diabetes on medication or those diagnosed for the first time with fasting blood glucose (FBG) ≥7.0mmol/l with glycated hemoglobin (HbA1c) ≥6.5%. Secondary (stress) hyperglycemia was defined as fasting blood glucose ≥7.0mmol/l but with glycated hemoglobin ≤6.5%. Normal glycaemia was defined as a blood glucose <7.0mmol/l in a patient without a prior history of diabetes [23].

Kidney dysfunction was defined as having a serum creatinine ≥133µmol [24]. Elevated liver enzymes have been associated with cardio-metabolic diseases such as metabolic syndrome, hypertension, and cardiovascular diseases [25,26]. We defined elevated bilirubin as ≥21µmol/l, alkaline phosphatase (ALP) ≥130IU/L, γ-glutamyl transpeptidase (GGT) ≥55IU/L, ALT ≥45IU/L and AST ≥42IU/L [27]. For C-reactive protein (C-RP), levels less than 1 mg/dl were considered low risk while levels between 1 mg/dl and 3 mg/dl were considered a moderate risk, and a level greater than 3 mg/dl was considered high vascular risk [28]. The D-dimers which are markers of hypercoagulability and predictors of cardiovascular events [29] were categorized as high when ≥0.5mg/l [30]. Chest findings on computerized tomography (CT) scan were reported based on COVID-19 Reporting and Data System (CO-RADS) for standardized assessment of pulmonary involvement with CO-RADS 1 very low and CO-RADS 5 very high [31]. We defined clinical outcomes as discharge, need for ICU admission, composite outcome (ICU admission, mechanical ventilation or death) and hospital (CTU) death [2] occurring within the first 10 days of admission.

**Statistical analysis:** we checked data for completeness, coded and entered it into Epi Data Manager version 4.6, and exported to STATA version 14.0 (Stata Corp LLC, College Station, Texas, United States of America) for analysis. Continuous variables were described using the mean and standard deviation if they were normally distributed or median and interquartile range (IQR) if non-normally distributed. Categorical variables were presented as frequencies and percentages and were compared using Chi-square tests. We performed multivariable logistic regression to determine cardiovascular risk factors and clinical outcomes significantly associated with TyG index while controlling for confounders. Variables with a p-value of less than 0.2 at bivariate analysis and those known to affect TyG index from the literature were included in the multivariable analysis. Furthermore, we ran a multicollineality test between the included variables, and those with a variance inflation factor >10 were excluded. Missing data was handled by the imputation method. We presented the results of the regression analysis with adjusted odds ratios (AOR), a 95% confidence interval and a two-sided p-value of <0.05 was considered statistically significant.

**Ethical consideration:** since this was a retrospective cross-sectional study, a waiver of informed consent and approval of the study were granted by Mengo Hospital Research Ethics Committee (approval number MH/REC/93/12-2021). We obtained further approval from the Uganda National Council of Science and Technology for the study (registration number HS2627ES). To ensure confidentiality, we used codes for de-identified selected files.

## Results

**Demographics and Clinical Presentation:** out of 314 patients, 176 (56%, 95%CI 50.5-61.5) were females while 138 (44%, 95% CI 38.5 -49.5) were males. The mean ± SD age was 58.2 ± 16.82 years. The majority presented with cough 274 (87.3%), shortness of breath 258 (82.2%), and fever 228 (72.6%). More than half of the patients had an SPO2 of less than 90%. Majority of patients 207 (65.9%) had had their symptoms for longer than 7 days (Table 1). The mean ± SD systolic blood pressure was 134.81 ± 24.75 mmHg while the median of oxygen saturation was 89% (IQR: 83-93). Patients were admitted after 7 days (IQR: 4-12) and spent 6 days (IQR: 4-9) in our unit (Table 2). Regarding comorbidities, hypertension 144 (45.9%) and diabetes 104 (33.1%) were the most common comorbidities. The most used medications were anti-hypertensives 140 (44.6%) and dexamethasone 139 (44.3%) (Table 3). A large number of patients 119 (38.3%) did not have chest CT scan results. About 195 (61.7%) patients had chest CT scan results. Of these, 2 (0.69%) were normal, 28 (9.0%) had COVID-19 Reporting and Data System (CO-RADS)-1, 42 (13.5%), CORADS-2, 37 (11.9%) CORADS-3. Both CORADS-4 and 5 were found among 32 (10.30%) of the patients while severe CORADS-6 was found among 19 (6.1%) patients.

**Table 1:** clinical presentation of the COVID-19 patients admitted to Mengo Hospital COVID-19 Treatment Unit from December 2020 to August 2021 (N=314)

Variable	Yes, n (%)
**Symptoms**	
Cough	274 (87.3)
Fever	228 (72.6)
Shortness of breath	258 (82.2)
Sore throat	43 (13.7)
Anosmia	13 (4.1)
Desgeusia	9 (2.9)
General body weakness	255 (81.2)
Chest pain	146 (46.5)
Vomiting	86 (27.4)
Duration of symptoms ≥7 days	207 (65.9)
Temperature ≥ 37.50c	75 (23.9)
Oxygen saturation <90%	163 (51.9)
Pulse ≥ 100b/min	147 (43.0)
Kussmaul’s breathing	3 (1.0)

N: frequency; %: percentage

**Table 2:** mean and median clinical characteristics of the COVID-19 patients admitted to Mengo Hospital Treatment Unit from December 2020 to August 2021 (N=314)

Variable	Mean ± SD or median (IQR)
Age, years	58.2 ± 16.820
Systolic blood pressure, mmHg	134.81 ± 24.750
Diastolic blood pressure, mmHg	79.89 ± 15.079
Pulse, beats/min	98 (84 - 111)
Temperature, °C	36.6 (36.2 - 37.2)
Oxygen saturation, %	89 (83 - 93)
Time to admission, days	7 (4 - 12)
Length of stay, days	6 (4 - 9)

SD: standard deviation; IQR: interquartile range

**Table 3:** comorbidities and medicine use history among the COVID-19 patients admitted to Mengo Hospital Treatment Unit from December 2020 to August 2021 (N=314)

Variable	Yes, n (%)
Comorbidity	
Diabetes	104 (33.1)
Hypertension	144 (45.8)
Coronary heart disease	25 (8.0)
Chronic liver disease	2 (0.6)
Chronic kidney disease	4 (1.3)
Stroke	13 (4.1)
HIV/AIDS	17 (5.4)
Cancer	8 (2.6)
Medicine	
Dexamethasone	139 (44.3)
Metformin monotherapy	38 (12.1)
Metformin + sulphonyl urea	26 (8.3)
Insulin monotherapy	39 (12.4)
Insulin + oral hypoglycemics	12 (3.8)
Lipid-lowering agents	72 (22.9)
Anti - hypertensives	140 (44.6)
ARV	16 (5.1)

n: frequency; HIV: human immunodeficiency virus; AIDS: acquired immune deficiency syndrome, ARV: antiretroviral drugs

**Figure 1:**
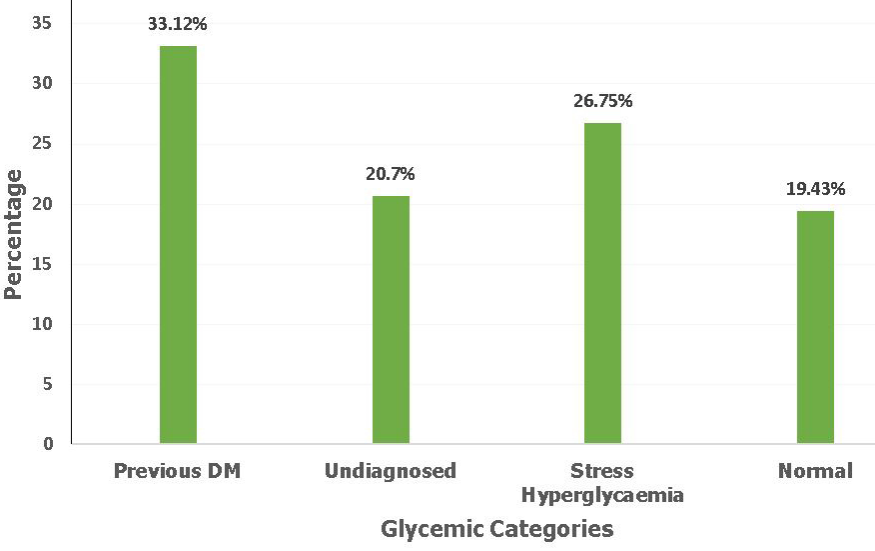
glycemic categories of COVID-19 patients admitted to the Mengo Hospital COVID-19 Treatment Unit from December 2020 to August 2021 (N=314)

**Figure 2:**
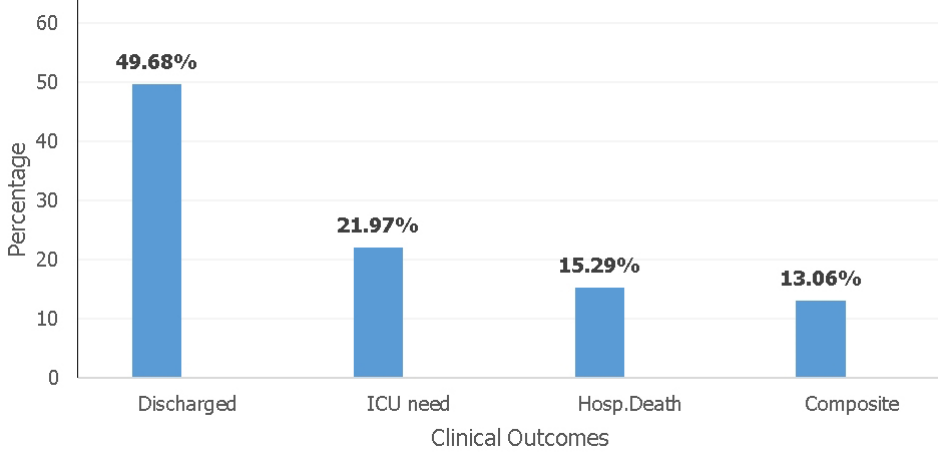
clinical outcomes of patients within 10 days of admission to the Mengo Hospital COVID-19 Treatment Unit from December 2020 to August 2021 (N=314)

**Laboratory findings, triglyceride-glucose index, vascular risk factors and clinical outcomes:** the mean LDL was 3.928 mmol/l ± 1.042 while the median total cholesterol was 5.325mmol/l (IQR: 4.2-6.28), triglycerides 2.04mmol/l (IQR: 1.58-2.9), fasting blood glucose 10.35mmol/l (IQR: 7.5-16.2) and C-RP14.98mg/dl (IQR: 7.8-29). The median TyG index was 9.76 (9.29-10.33) (Table 4). Out of 314 patients, a low TyG index of <9.04 was found among 46(14.6%, 95% CI: 0.111-0.190) while a high TyG index of ≥9.04 was found among 268 (85.4%, 95% CI: 0.809-0.889). Regarding vascular risk factors, the majority of patients (53.82%) were younger than 60 years. The majority of patients had elevated total cholesterol 174 (55.4%), triglycerides 222 (70.7%), and LDL 203 (64.7%) (Table 5). Out of 314 patients, 253 (80.6%) had elevated blood glucose levels. Out of these, 104 (33.1%) had a prior history of diabetes, 65 (20.7%) had undiagnosed diabetes, and 84 (26.8%) had stress-related hyperglycemia (Figure 1). One hundred and thirty-five patients (43%) had elevated systolic blood pressure whereas 78 (24.8%) had high diastolic blood pressure. Within the 10 days of admission, 156 (49.7%) of patients were discharged while 69 (22.0%) needed admission to ICU, 48 (15.3%) died in the unit and 41 (13.0%) had a composite outcome (Figure 2).

**Table 4:** mean and median laboratory findings of the COVID-19 patients admitted to Mengo Hospital Treatment Unit from December 2020 to August 2021 (N=314)

Variable	Mean ± SD or median (IQR)
White blood count, x10^9^/l	8.83 (6.06-12)
Neutrophil count, x10^9^/l	6.83 (4.08-10.31)
Lymphocyte count, x10^9^/l	1.1 (0.84-1.48)
Platelet count, x10^9^/l	207.5 (159-283)
Fasting blood glucose, mmol/l	10.35 (7.5-16.2)
Glycated hemoglobin, %	6.5 (4.8 - 9.1)
D-dimers, mg/l	1.46 (0.65 - 3.54)
C-reactive protein, mg/dl	14.98 (7.8 - 29)
Erythrocyte sedimentation rate, ml/min	45 (18 - 70)
Sodium, mmol/l	135 (132 - 138)
Potassium, mmol/l	4.6 (4.14-4.98)
Creatinine, mmol/l	89.6 (71 - 118)
Total bilirubin, µmol/l	7.15 (5 - 13.8)
Alanine amino transaminase, IU/L	33 (22.7-50.7)
Aspartate amino transaminase, IU/L	39 (28-67.6)
Alkaline phosphatase, IU/L	112 (85-138)
ƴ-glutamyl transpeptidase, IU/L	60 (38-98)
Total cholesterol, mmol/l	5.325 (4.2 - 6.28)
High density lipoprotein cholesterol, mmol/l	1.08 ± 0.84
Low density lipoprotein cholesterol, mmol/l	3.928 ± 1.0418
Non-high-density lipoprotein cholesterol, mmol/l	4.25 (3 - 5.13)
Triglycerides, mmol/l	2.04 (1.58 - 2.9)
Triglyceride glucose index	9.76 (9.29 - 10.33)

SD: standard deviation; IQR: interquartile range

**Table 5:** vascular risk factors among the COVID-19 patients admitted to Mengo Hospital Treatment Unit from December 2020 to August 2021 (N=314)

Variable		n (Frequency)	% (percentage)
Fasting blood glucose	<7mmol/l	69	22.0
	≥7mmol/l	245	78.0
Glycated hemoglobin	<6.5%	154	49.0
	≥6.5%	160	51.0
Systolic blood pressure	<140mmHg	179	57.0
	≥140mmHg	135	43.0
Diastolic blood pressure	<90mmHg	236	75.2
	≥90mmHg	78	24.8
Total cholesterol	<5.17mmol/l	140	44.6
	≥5.17mmol/l	174	55.4
Triglycerides	<1.68 mmol/l	92	29.3
	≥1.68mmol/l	222	70.7
High density lipoprotein cholesterol	>1.3	176	56.0
	<1.3mmol/l	138	44.0
Low density lipoprotein cholesterol	<4.1mmol/l	111	35.3
	≥4.1mmol/l	203	64.7
Alanine amino transaminase	<45IU/L	191	60.8
	≥45IU/L	123	39.2
Aspartate amino transaminase	<42IU/L	166	52.9
	≥42IU/L	148	47.1
Erythrocyte sedimentation rate	<20ml/hr	68	21.7
	≥20ml/hr	246	78.3

n=Frequency, %: Percentage

**TyG association with vascular risk factors and clinical outcomes:** the vascular risk factors that were independently associated with TyG index were glycated hemoglobin (AOR=1.029, 95%CI 0.561-1.496, P<0.001), low-density lipoprotein cholesterol (AOR=0.121, 95%CI 0.023-0.219, P=0.016), high-density cholesterol (AOR=1.956, 95%CI 1.299-2.945, P=0.001), total cholesterol (AOR=2.177, 95%CI 1.5222-3.144, P<0.001). Among the clinical outcomes, TyG index was significantly associated with hospital death (AOR=0.778, 95%C 10.623-0.972, P=0.028), composite outcome (AOR=1.823, 95% CI 1.221-2.559, P=0.023). There was no association between systolic blood pressure (AOR=0.179, 95% CI -0.412-0.712, P=0.496, diastolic blood pressure (AOR=0.168, 95%CI -0.332-0.657, P=0.514), non-high-density cholesterol (AOR=2.066, 95%CI 0.709-5.948, P=0.185) and CT scan findings (AOR=1.160, 95%CI 0.710-1.897, P=0.553) with TyG index (Table 6).

**Table 6:** bivariate and multivariate analysis of triglyceride-glucose index against cardiovascular risk factors and clinical outcomes among the COVID-19 patients admitted to Mengo Hospital Treatment Unit from December 2020 to August 2021 (N=314)

Variable	Unadjusted odds ratio (95% CI)	P-value	Adjusted odds ratio (95% CI)	P-value
**Systolic blood pressure**				
<140mmHg	1		1	
≥140mmHg	0.158(-0.315-0.622)	0.521	0.179(-0.412-0.0.712)	0.496
**Diastolic blood pressure**				
<90mmHg	1		1	
≥90mmHg	0.145(-0.263-0.553	0.485	0.168(-0.332-0.657	0.514
**Glycated hemoglobin**				
<6.5%	1		1	
≥6.5%	1.039(0.537-1.54)	<0.001	1.029(0.561-1.496)	<0.001
**Low-density cholesterol**				
<2.82mmol/l	1		1	
≥2.82mmol/l	0.129(0.023-0.235)	0.017	0.121(0.023-0.219)	0.016
**Non-high-density cholesterol**				
<3.33mmol/l	1		1	
≥3.33mmol/l	2.438(-0.381-5.260)	0.09	2.066(0.709-5.948)	0.185
**High-density cholesterol**				
≥1.3mmol/l	1		1	
<1.3mmol/l	1.587(1.056-2.85)	0.026	1.956(1.299-2.945)	0.001
**Total cholesterol**				
<5.13 mmol/l	1		1	
≥5.13mmol/l	1.599(1.411-1.811)	<0.001	2.177(1.522-3.144)	<0.001
**Hospital death**				
No	1		1	
Yes	1.141(0.921-1.415)	0.025	0.778(0.623-0.972)	0.029
**Composite outcome**				
No	1		1	
Yes	1.136(0.452-2-888)	0.034	1.823(1.22-2.559)	0.021
**CT scan findings**				
Normal	1		1	
≥CORADS 1	2.82(1.71-4.666)	<0.001	1.160(0.710-1.897)	0.553

CO-RADS: COVID-19 reporting and data system; CT: computerized tomography; CI: confidence interval

## Discussion

The current study was conducted to establish the association between the triglyceride-glucose index and vascular risk factors and clinical outcomes among adult COVID-19 patients admitted to Mengo Hospital. The study showed an association between TyG index and vascular risk factors and clinical outcomes among COVID-19 patients admitted to Mengo Hospital. TyG index was associated with TC, LDL, HDL-c, HbA1c, hospital death, and composite outcome ((ICU admission, mechanical ventilation, or death). Hypertension, non-HDL, and chest CT findings were not significantly associated with TyG index.

Our study showed TyG index (a marker of insulin resistance) was associated with HbA1c. More than 80% of our patients were hyperglycemic with more than half of this proportion comprised of undiagnosed diabetes and stress hyperglycemia. Elevated HbA1c was found among those with pre-existing and undiagnosed diabetes. Elevated HbA1c denotes hyperglycemia among these patients for the previous 3 months before they were diagnosed with COVID-19. Among patients with diabetes, one of the pathological defects is insulin resistance which may occur in an individual as part of the metabolic syndrome [5]. Before deterioration of the insulin-resistant state into overt diabetes, there is ensuing hyperinsulinemia [5] which is linked with chronic activation of pro-inflammatory signaling pathways and immune dysregulation [32]. Additionally, exposure of macrophages to high levels of insulin leads to the production of pro-inflammatory cytokines [2]. In critical illness such as severe SARS-CoV-2 infection, there is profound inflammatory dysregulation with the production of excessive levels of inflammatory cytokines (cytokine storm) [2]. This potentiates the effects of a subclinical inflammatory state which is sometimes found in people with diabetes. In our study, more than 50% of patients had longstanding hyperglycemia with 33.12% with a previous history of diabetes and 20.7% with undiagnosed diabetes. Stress hyperglycemia that could have been due to the critical SARS CoV-2 infection was found in only 26.75% of the patients. This means the majority of our patients were already predisposed to hyperglycemia prior to COVID-19 disease.

One of the hallmarks of insulin resistance is dyslipidemia; patients with metabolic syndrome have hypertriglyceridemia, and low levels of HDL [5]. In our study, the majority of patients had hyperlipidemia characterized by elevated levels of TC, TG, non-HDL, and LDL and these constitute key vascular risk factors. Our findings are similar to those demonstrated in an Italian study by Bellia A *et al*. 2021 who found highly prevalent atherogenic dyslipidemia in patients admitted with critical COVID-19. The same study found a significant association between atherogenic dyslipidemia and poor outcomes most especially hospital death [33]. We showed a significant association between TyG index and some of these components of dyslipidemia most especially total cholesterol and HDL.

An elevated TyG index denotes a high cardiovascular disease (CVD) risk. In our study, the median TyG index was 9.76 (9.29-10.33) and 88.4% of the patients had a high TyG index denoting a high CVD risk among our patients. A high TyG index is a reliable predictor of cardiovascular events namely coronary artery disease (CAD, premature CAD and adverse outcomes in patients with CAD [34]. A high TyG index does not only correlate with CVD risk factors but also underscores the severity and adverse clinical outcomes among patients with COVID-19 [20]. Our study showed a high TyG index was associated with hospital death and composite outcomes among our patients. There is a high propensity of COVID-19-related complications among individuals with a high CVD risk and cardiovascular disease [1]. A study by Ren *et al*. showed that TyG index predicted severe cases and mortality of COVID-19 patients [12]. The same study showed that TyG index was significantly higher in the severe cases and death groups. Unlike our study, there was no association between TyG index and vascular risk factors probably due to a smaller sample size in the Chinese study. Similarly, a Mexican observation cohort study showed that biomarkers of insulin resistance such as elevated triglycerides and low HDL predict severe complications of COVID-19 such as the need for mechanical ventilation and hospital death [35]. These findings are similar to our findings despite differences in study design and sample size.

Despite a significant number of our patients (45.9%) having a positive history of hypertension, we did not show a relationship between systolic or diastolic pressure and TyG index probably because the majority of our patients had well-controlled systolic and diastolic blood pressure lower than 140mmHg and 90mmHg respectively. It is worth noting the mean systolic and diastolic blood pressure in our study were 134.81mmHg ± 24.75 and 79.89mmHg ± 15.079 respectively underscoring the fair control of this vascular risk factor in our patients. Our findings are not consistent with one study by Zhu B *et al*. where elevated TyG index was significantly associated with hypertension [36]. We note the significant differences between this study and ours, the Chinese study was population-based with a larger sample size while ours was hospital-based with younger patients with a mean age of 58 years compared to 65 years in the Chinese study [36]. Hypertension alters the delivery of insulin and glucose to skeletal cells leading to impaired glucose uptake. Hypertension may potentially impair vasodilation of skeletal muscles due to related vascular structural changes and rarefaction [37].

Our study showed that critically ill patients have high levels of insulin resistance that is associated with a number of vascular risk factors and poor clinical outcomes. Screening for these vascular risk factors and insulin resistance among critically ill patients may help in planning care and prognostication of patients.

A number of limitations of this study are worth noting; it was a retrospective study so not all relevant information and data could be obtained for all patients. Secondly, we did not have data on pre-infection variables such as lipid profile so the effect of COVID-19 on these parameters could not be ascertained. Thirdly, this was a private single-center study recruiting middle-income patients; findings may not be generalizable to other facilities most especially the public ones. Additionally, we did not analyze data on non-hospitalized patients and the public to compare the outcomes. Despite these limitations, we had a robust data set on glycated hemoglobin, lipid profile, and other laboratory parameters to provide a better understanding of the association between TyG index and vascular risk factors and clinical outcomes among COVID-19 patients. To our knowledge, this is the first study to generate data on the association between insulin resistance and vascular risk factors and clinical outcomes among critically ill patients in Uganda.

## Conclusion

A high TyG index was associated with vascular risk factors and poor clinical outcomes among COVID-19 patients. Screening for these vascular risk factors and insulin resistance among critically ill patients may aid in planning care and prognostication of patients.

### What is known about this topic

- *TyG index is a simple and reliable surrogate marker of insulin resistance;*
- *TyG index is a predictor of cardiovascular disease;*
- *TyG index is associated with adverse clinical outcomes among critically ill COVID-19 patients.*

### What this study adds

- *Insulin resistance is highly prevalent among critically ill COVID-19 patients in Uganda;*
- *Insulin resistance is not associated with hypertension among critically ill patients in our setting.*
